# Coping as a Moderator for the Relationship Between Perceived Stress and Satisfaction with Life in the Group of Uniformed Personnel Treated in Mental Health Clinic

**DOI:** 10.3390/jcm14176225

**Published:** 2025-09-03

**Authors:** Mateusz Curyło, Michał Zabojszcz, Lidia Tkaczyk, Jaromira Iwolska, Marcin Mikos, Łukasz Strzępek, Aleksandra Czerw, Olga Partyka, Monika Pajewska, Mariola Głowacka, Adrianna Frydrysiak-Brzozowska, Zofia Sienkiewicz, Grażyna Dykowska, Katarzyna Sygit, Oleh Lyubinets, Izabela Gąska, Magdalena Konieczny, Elżbieta Grochans, Anna M. Cybulska, Daria Schneider-Matyka, Ewa Bandurska, Weronika Ciećko, Jarosław Drobnik, Piotr Pobrotyn, Dorota Waśko-Czopnik, Julia Pobrotyn, Adam Wiatkowski, Michał Marczak, Petre Iltchev, Remigiusz Kozlowski

**Affiliations:** 1 Institute of Health Sciences, University of the National Education Commission, 30-084 Krakow, Poland; 2 Psychotherapy Day-Care Unit, Hospital of the Ministry of Interior and Administration in Krakow, 30-053 Krakow, Poland; 3 Institute of Medical Sciences, Collegium Medicum, Jan Kochanowski University, 25-317 Kielce, Poland; 4 Department of Bioinformatics and Public Health, Andrzej Frycz Modrzewski Krakow University, 30-705 Krakow, Poland; 5 Department of Surgery, Andrzej Frycz Modrzewski Krakow University, 30-705 Krakow, Poland; 6 Clinical Department of General and Oncological Surgery, Saint Raphael Hospital, 30-693 Krakow, Poland; 7 Department of Health Economics and Insurance, Medical University of Warsaw, 00-581 Warsaw, Poland; 8 Department of Economic and System Analyses, National Institute of Public Health NIH-National Research Institute, 00-791 Warsaw, Poland; 9 Nursing Department, Faculty of Health Science, Institute of Nursing, Collegium Medicum, Masovian University in Płock, 09-402 Płock, Poland; 10 Department of Nursing, Social and Medical Development, Medical University of Warsaw, 01-445 Warsaw, Poland; 11 Institute of Nursing, College of Engineering and Health, 02-366 Warsaw, Poland; 12 Faculty of Medicine and Health Sciences, University of Kalisz, 62-800 Kalisz, Poland; 13 Department of Psychiatry and Psychotherapy, Danylo Halytsky Lviv National Medical University, 79010 Lviv, Ukraine; 14 Medical Institute, Jan Grodek State University in Sanok, 38-500 Sanok, Poland; 15 Department of Nursing, Faculty of Health Sciences, Pomeranian Medical University in Szczecin, 71-210 Szczecin, Poland; 16 Center for Competence Development, Integrated Care and e-Health, Medical University of Gdansk, 80-204 Gdansk, Poland; 17 Department of Family Medicine, Faculty of Medicine, Wroclaw Medical University, 51-141 Wroclaw, Poland; 18 Citodent Dental Center, Furtak-Pobrotyn & Company Limited Partnership, 05-220 Olawa, Poland; 19 Department of Gastroenterology, Hepatology with Inflammatory Bowel Disease Subunit, Provincial Specialist Hospital J. Gromkowskiego, 51-149 Wroclaw, Poland; 20 Department of Non-Surgical Clinical Sciences, Faculty of Medicine, Wrocław University of Science and Technology, 50-370 Wroclaw, Poland; 21 Faculty of Medicine, Wroclaw Medical University, 50-345 Wroclaw, Poland; 22 Department of Innovation, Merito University in Poznan, 61-895 Poznan, Poland; 23 Department of Management and Logistics in Healthcare, Medical University of Lodz, 90-131 Lodz, Poland

**Keywords:** stress, satisfaction with life, mental health

## Abstract

**Background/Objectives:** Uniformed personnel are highly exposed to occupational stress, which increases the risk of mental health problems. This study examined whether coping styles moderate the relationship between perceived stress and satisfaction with life among uniformed personnel treated for bodily distress disorder or post-traumatic stress disorder. **Methods:** A cross-sectional study was conducted with 183 participants (81% male, aged 30–66 years). Standardized questionnaires were administered: the Perceived Stress Scale (PSS-10), Satisfaction with Life Scale (SWLS), and Coping Inventory for Stressful Situations (CISS). Pearson correlations with 95% confidence intervals were computed, and moderation analyses were conducted using separate regression models for each coping style with bootstrap estimation (1000 samples). Gender differences were examined using *t*-tests with Cohen’s d. **Results:** Perceived stress was negatively correlated with life satisfaction (r = −0.43, 95% CI [−0.54, −0.29], moderate effect). Emotion-oriented coping correlated negatively with life satisfaction (r = −0.28, 95% CI [−0.42, −0.14]), while social diversion correlated positively (r = 0.21, 95% CI [0.07, 0.35]). Women reported higher stress (Cohen’s *d* = 0.60) and lower life satisfaction (Cohen’s *d* = −0.50) than men. Moderation analysis revealed that emotion-oriented coping significantly intensified the negative effect of stress on life satisfaction (*B* = −0.01, *p* = 0.019). **Conclusions:** Perceived stress strongly impairs life satisfaction in uniformed personnel, particularly among those relying on emotion-oriented coping. Targeted interventions, such as emotion regulation training, mindfulness, and cognitive restructuring, may enhance resilience and mitigate stress-related declines in well-being in this high-risk occupational group.

## 1. Introduction

Stress is the experience of disruption to homeostatic balance, both physiological and psychological [1]. It appears when excessive demands are placed on the body and the mind. If chronic, stress can have serious consequences. Perceived stress is a cognitive concept underlying the subjective point of view on the stress level from [2]. The same situation can be assessed as more or less stressful depending on a person, because it can be perceived as more or less unpredictable, uncontrollable, or overwhelming.

Uniformed personnel are a unique occupational group because their professional activity involves exposure to primary and secondary trauma [3]. Tensions surrounding even common tasks need specific coping strategies [4]. The uniformed personnel are in a situation of increased risk of developing mental disorders due to the stressful working environment [5]. A study concluded on a sample of soldiers in the US army revealed that 25.1% of respondents met criteria for any 30-day disorder and 11.1% for multiple disorders [6]. Still, there is a literature gap regarding the topic. For example, the search performed with the use of the EBSCOhost platform [7] with the use of keywords “uniformed personnel” and “mental health diagnoses” performed in August 2025 revealed only 10 relevant papers.

One of the consequences of chronic stress or strain can be developing bodily distress disorder (6C20), which is characterized by the presence of bodily symptoms that are distressing and drawing excessive attention to their nature and progression. These symptoms are persistent, i.e., are present on most days. The problem lasts for at least several months. Multiple bodily symptoms are experienced. However, they may vary over time. It is also possible that there exists a single symptom, usually a pain or fatigue. The symptoms, the associated distress, and paying close attention to both have an effect on the individual’s functioning [8]. Another mental disorder associated with being a member of uniformed personnel is post-traumatic stress disorder (6B40) that develops following exposure to extremely threatening or horrific events [9]. It is characterized by re-experiencing the traumatic events in the form of vivid, persistent memories, flashbacks, or nightmares, usually with strong or overwhelming emotions and even physical sensations [8]. Another cluster of symptoms is characterized by avoidance of thoughts and memories of the events, which leads to avoidance of activities, situations, or people who can remind one of the traumatic events. Also, persistent perceptions of a heightened current threat are present. The symptoms of PTSD last for at least several weeks and cause impairment in one’s functioning. A phenomenon accompanying PTSD can be alexithymia, which is the loss of the ability to understand and name emotions, according to Wojciechowska et al. [10].

Specific psychological models are developed in order to train appropriate coping [11,12]. Coping is defined as individual efforts to manage distressing problems and emotions that affect the physical and psychological outcomes of stress [1]. It is a set of behaviors and cognitive processes that are an intermediate between events causing stress and the stress consequences. A coping style is a set of typical ways of confronting a stressful situation in order to deal with it [13]. According to Endler [14], there are three basic coping styles: task-oriented, emotion-oriented, and avoidance-oriented. Task-oriented coping involves efforts aimed at solving the current problem. Emotion-oriented coping consists of emotional reactions to the stressful situation. Avoidance-oriented coping entails behaviours and cognitions aimed at avoiding the stressful situation. It can be either being involved in distraction or in social diversion. Endler describes task-oriented coping as most efficacious in situations allowing for control to be exerted. Emotion-oriented coping can be more effective in uncontrollable situations. Avoidance-oriented coping may initially be appropriate as a reaction to stress. Task-oriented coping is most efficacious in the long run. The intensity of coping styles, the regular cognitions, and behaviors of responding to stressful events affect both the level of the stress perceived, anxiety, somatic complaints, and, in the long run, satisfaction with life. Even though the Endler framework was introduced in the 1990s, it is still in use in the current research. The role of emotion-oriented coping, task-oriented coping, and avoidance was investigated recently in the context of adjusting to the COVID-19 pandemic [15,16]. Another example of recent use of the Endler approach is the field of studying differences between different professional groups regarding coping styles [17]. Individual differences between army officers, executives, and freelance workers in coping styles and stress levels were found. Also, the older people within these groups were found to cope with stress better by choosing an appropriate strategy.

Within the framework of Lazarus and Folkman [18], appraisals of both the threat and one’s resources are the basis of distress. If the perceived threat exceeds the resources, the experienced distress is elevated. The resources in this model include personal resources, like coping styles or other personality factors. Specifically, emotion-oriented coping can be relevant. Higher intensity of emotion-oriented coping may involve a lower level of perceived ability to cope. However, other coping styles may also be important. Related to the appraisal of one’s resources, they may moderate the relationship between the perceived level of stress and one’s well-being. The framework of Lazarus and Folkman is also a basis for further research. For example, task efficacy, one’s belief in their task handling capabilities, was found to be an additional moderator to the mediating role appraisals play [19]. The cognitive appraisals were found to be mediators of the relationship between stress and general mental health in a large sample of medical personnel [20].

Satisfaction with life also refers to a cognitive, judgmental process of assessing one’s life quality, since it is different from positive and negative affect [21]. It is also a concept referring to the subjective point of view. The assessment needs to be compared to an appropriate standard that people set for themselves. For example, health, energy, and prosperity can be desirable for the majority. However, there are individual differences regarding the importance of such values.

The higher level of perceived stress was found to co-occur with lower satisfaction with life, specifically if professional activity involves greater exposure to stress [22]. The correlation between perceived stress and satisfaction with life was found to be stronger than the correlations between coping styles and satisfaction with life [23]. That is not to say that coping styles and strategies are not related to satisfaction with life, but perhaps they play a different role. Rather than predictors, they can be analyzed as moderators for the relationship between perceived stress and satisfaction with life. Therefore, we formulated the following hypotheses:

**H1.**  *The level of perceived stress correlates negatively with satisfaction with life.*

**H2.**  *Coping styles are related to satisfaction with life; however*, *the relationships between coping styles and satisfaction with life are weaker than the relationship between the level of perceived stress and satisfaction with life*.

**H3.**  *Coping styles are moderators of the relationship between the level of perceived stress and satisfaction with life*.

## 2. Materials and Methods

A group of 183 participants aged 30–66 (*M* = 44.72; *SD* = 5.84) participated in the current study. The participants were all uniformed personnel members treated in a mental health clinic and undergoing outpatient treatment from February 2024 to August 2024. The patients who participated were diagnosed with bodily distress disorder (6C20) or post-traumatic stress disorder (6B40). No other criteria were applied. The duration of therapy was 5 to 6 weeks. Out of 193 patients satisfying the inclusion criteria, 10 patients (5.2%) refused to participate.

The current sample size allowed us to detect the effect size of the value equal to at least 0.04 in terms of Cohen’s *f*^2^ effect size measure to be statistically significant regarding the moderation effects verified below. The effect size of 0.04 is stronger than a small effect size, which, according to Cohen, is equal to 0.02 [24], but weaker than 0.15, which, according to Cohen, is a medium effect size.

Table 1 depicts sociodemographic characteristics of the current sample.

The majority of participants were male. Also, the majority of participants completed higher education. All participants filled out psychological questionnaires at the same time point. The order of questionnaires was CISS, SWLS, and PSS-10. The surveys were ordered by the Hospital of the Ministry of Interior and Administration in Krakow for the day psychotherapy ward, where this study was conducted. Data collection was performed by a qualified psychologist working in the entity. This study obtained the consent of the bioethics committee, the consent of the director of the medical facility to conduct this study on its premises, and written consents of patients collected by the psychologist, which, together with the questionnaires, are kept in the archives of the Hospital of the Ministry of Interior and Administration in Krakow.

### 2.1. Coping Inventory for Stressful Situations

Coping styles were assessed with the Polish version [25] of the Coping Inventory for Stressful Situations (CISS) originally developed by Endler and Parker [26]. The inventory is based on 48 diagnostic items and measures three types of coping: emotion-oriented, task-oriented, and avoidant, 16 items for each of the three. The avoidant style has two subscales: distraction and social diversion. Each item is based on a 5-point scale (from 1 = Never to 5 = Always). The specific items for specific types of coping are scattered throughout the questionnaire and do not form any patterns. The Cronbach alpha coefficients for the Polish version fall within the range 0.72–0.92.

### 2.2. Perceived Stress Scale

PSS-10 (Perceived Stress Scale) questionnaire [27] was used for measuring perceived stress intensity. It consists of 10 questions, each scored from 0 (never) to 4 (very frequently). Four items need to be reversed before calculating the total score. The sum of scores for the items falls within the range of 0–40 points. The higher the score, the higher the level of perceived stress. The Cronbach alpha coefficient for the Polish version was equal to 0.86. Test–retest reliability was equal to 0.90 when tested after two days and to 0.72 when tested after four weeks.

### 2.3. Satisfaction with Life Scale

The SWLS (Satisfaction with Life Scale) questionnaire was used for measuring life satisfaction [28]. This questionnaire consists of 5 questions, each scored from 1 (completely disagree) to 7 (completely agree). The sum of scores for the answers gives the total score within the range of 5–35 points. The higher the score, the higher the level of life satisfaction. The Cronbach alpha coefficient for the Polish version was equal to 0.81. Test–retest reliability was equal to 0.86 when tested after six weeks.

For the data analysis, firstly, descriptive statistics were calculated, and deviations from normal distribution were assessed. Also, Pearson correlation coefficients between all analyzed variables were calculated. All coping styles were analyzed as potential moderators of the relationship between perceived level of stress and satisfaction with life. For this purpose, a moderation analysis was applied. Each coping style was analyzed in a separate moderation model. The bootstrap method with 1000 samples was used for assessing statistical significance. Moderation analysis was performed with the use of Jamovi 2.6.26 software [29] with the additional module medmod 1.1.0 installed. This module automatically handles mean centering. Additionally, the correlation analysis and the analysis of the differences between female and male participants were provided. We assessed the statistical significance of the differences with the use of an independent sample *t*-test and calculated Cohen’s *d* effect size measure [24]. Cohen’s *d* effect size measure was interpreted following the guidelines provided by the author, i.e., small (*d* = 0.2), medium (*d* = 0.5), and large (*d* = 0.8).

## 3. Results

### 3.1. Descriptive Statistics

We started with the preliminary analysis. Table 2 depicts descriptive statistics for the analyzed interval variables, i.e., mean values, standard deviations, minimum and maximum values, measures of skewness and kurtosis, and the values of Shapiro–Wilk for normality. Also, the values of Cronbach’s alpha reliability coefficient are provided.

The distributions of task-oriented coping, emotion-oriented coping, avoidant coping, and social diversion significantly differed from the normal distribution. All these distributions were negatively skewed. The values of reliability coefficients for all analyzed variables were satisfactory.

### 3.2. Correlation Analysis

Next, we analyzed correlations between all analyzed variables, including participants’ age. The results with 95% confidence intervals are depicted in Table 3.

The level of perceived stress correlated negatively with satisfaction with life, which confirms hypothesis H1. The relationship strength was moderate.

Emotion-oriented coping correlated with satisfaction with life, and negatively avoidant coping, specifically social diversion, correlated with satisfaction with life positively. However, these relationships were weak, which is consistent with hypothesis H2.

Task-oriented coping was positively associated with emotion-oriented coping (moderately), avoidant coping (moderately), distraction seeking (weakly), and social diversion (moderately). The correlation with social diversion was strongest. Emotion-oriented coping was also associated positively with avoidant coping (weakly) and its dimensions, i.e., distraction seeking (weakly) and social diversion (weakly). The level of perceived stress was positively related to emotion-oriented style (moderately) and negatively related to social diversion (weakly). There was no statistically significant association between participants’ age and any other analyzed variable.

### 3.3. Differences Between Female and Male Participants

Also, we analyzed gender differences. Table 4 presents mean values of analyzed variables acquired in the group of female participants and in the group of male participants, with the values of the independent sample *t*-test and the values of Cohen’s *d* effect size measure.

The level of perceived stress was significantly higher in the group of women, while the level of satisfaction with life was significantly higher in the group of men. The effect size of both effects was average. In the analyses performed by the authors of Polish adaptations of the questionnaires, no such differences were detected. This applies to both perceived stress [27] and satisfaction with life [28]. Therefore, these differences may be interpreted as characteristic of the population of uniformed personnel diagnosed with bodily distress disorder or post-traumatic stress disorder, and for the general populations of females and males.

### 3.4. Moderation Analysis

In the main analysis, each of the coping styles was analyzed as a moderator of the relationship between the level of perceived stress and satisfaction with life. The coping styles were analyzed separately in a separate statistical model. The significance of perceived stress, the coping style, and the interaction between the two was analyzed in each model. Table 5 presents the regression coefficients acquired.

**Figure 1:**
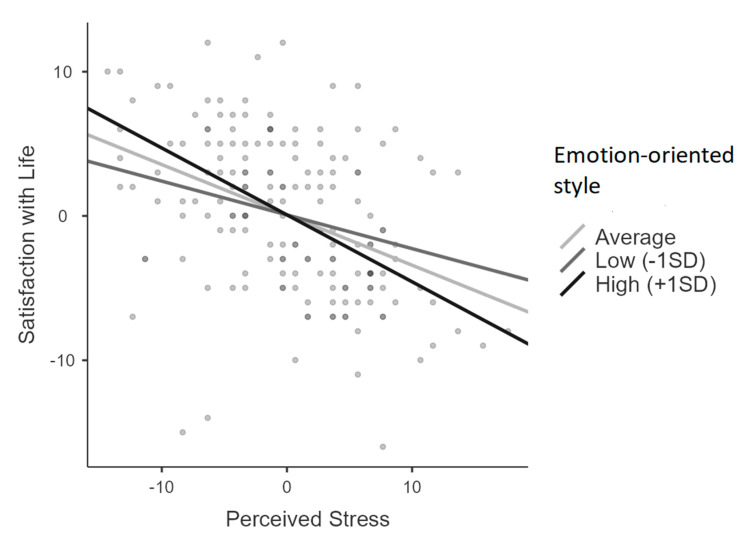
Relationship between the level of perceived stress and satisfaction with life at three levels of emotion-oriented coping.

In all five moderation models, the level of perceived stress was negatively related to satisfaction with life. None of the five coping styles was related to satisfaction with life significantly when the level of perceived stress was controlled for. One statistically significant moderation effect was detected. Emotion-oriented coping was a moderator of the relationship between the level of perceived stress and satisfaction with life. The negative relationship between the level of perceived stress and satisfaction with life was weaker if the level of emotion coping was lower (see Figure 1). The regression coefficients for the relationship were equal to *B* = −0.23, *Z* = −3.16, *p* = 0.002, if level of emotion-oriented coping was at −1 *SD*, i.e., 31.13, to *B* = −0.35, *Z* = −5.89, *p* < 0.001, if level of emotion-oriented coping was average and to *B* = −0.46, *Z* = −5.46, *p* < 0.001, if level of emotion-oriented coping was at +1 *SD*, i.e., 54.07. The statistically significant moderation effect is consistent with hypothesis H3.

## 4. Discussion

The results of the current study confirmed that the level of perceived stress correlated negatively with satisfaction with life. Also, emotion-oriented coping was negatively correlated with satisfaction with life, and avoidant coping, specifically social diversion, correlated with satisfaction with life positively, and these correlations were weaker than the correlation between perceived stress and satisfaction with life. What is more, the relationships between the coping styles and satisfaction with life were insignificant when controlling for the level of perceived stress. Out of the five coping styles analyzed, emotion-oriented coping was the only moderator of the relationship between perceived stress and satisfaction with life. The higher the level of emotion-oriented coping, the stronger the negative relationship between the level of perceived stress and satisfaction with life. It seems that a higher level of perceived stress impacts satisfaction with life more strongly if the level of emotion-oriented coping is elevated as well. It is possible that maladaptive emotional regulation characteristic of bodily distress disorder (6C20) and post-traumatic stress disorder (6B40) strengthens internalization of stress and, at the same time, reduces perceived control, which leads to a higher impact of perceived stress on well-being.

The results acquired by Barański and Poprawa [30] on a large sample of 622 participants suggest that the role of task-oriented coping is crucial, because it is related to both a higher level of satisfaction with life and lower frustration, while escape–avoidance coping is related to a higher level of stress, lower level of satisfaction with life, and higher frustration. However, this study was conducted on a sample from the general population, and the current study is based on a sample of uniformed personnel members treated in a mental health clinic and undergoing outpatient treatment diagnosed with bodily distress disorder (6C20) or post-traumatic stress disorder (6B40). Therefore, it seems that the role of various coping styles can be different depending on the current situation of participants, including their mental health status.

In a study conducted on a sample of 300 male police personnel [31], it was found that adaptive coping had a significant moderating effect on the relationship between stress and mental health for inspectors and officers, and the active coping strategies had a partial moderating effect on the relationship between stress and mental health in inspectors. These results also show that the specific coping strategies play the role of moderators depending on the current situation of participants, the rank involving a different set of duties in this case.

The results of the study conducted on a sample of 387 military academy cadets [32] revealed that hardiness, which is a sense of commitment and purpose in life, but also a belief that control or influence on outcomes is feasible, was a moderator for the relationship between the level of stress and health and eating disorder symptoms. The authors conclude that the symptoms can be reduced through programs aimed at stress coping strategies.

On the other hand, the study conducted on a sample of 104 law enforcement officers from various criminal justice agencies [33] revealed that if the level of active coping was average or high and, at the same time, perceived organizational support was low or average, the level of operational stress was positively related to poor sleep quality, which shows that under specific conditions active coping may not be beneficial.

Emotion-oriented coping was found to be one of the most significant predictors of burnout in a sample of 4409 Italian military personnel [34]. However, in contrast to emotion-oriented coping from the framework of Endler and Parker, the concept of positive emotion-focused coping was developed [35]. It refers to accepting the realities of an uncontrollable situation, trying to find the positive meaning, and keeping a sense of humor. Positive emotion-focused coping is related negatively to PTSD symptoms [36]. Five studies mentioned above were based on the samples from non-clinical uniformed personnel populations. The results from these studies show the moderating role of active coping for the relationship between stress and well-being. Our study, on the other hand, suggests the moderating effect of emotion-oriented coping. A study conducted on a sample of 2347 teachers from 93 schools [37] investigated the relationship between the level of stress and job satisfaction, which is also a part of satisfaction with life. The results showed that an increased level of stress had a weaker negative impact on job satisfaction for participants who rated their coping higher compared to those with average or low coping ratings.

Our study has certain limitations. First, it is only a cross-sectional study, which makes assessing the possible dynamic of change and its impact impossible. Specifically, a study investigating how the change in coping affects the relationship between perceived level of stress and satisfaction with life would be valuable. In the cross-sectional design treatment, effects on coping strategies were not controlled. Our cross-sectional study prevents any causal inference about coping–stress relationships. Also, the current study is based on a specific sample of participants with specific diagnoses of mental disorders. A comparison with results acquired on an equivalent sample of participants without clinical symptoms currently elevated would definitely be beneficial. Our clinical sample limits generalizability to healthy uniformed personnel. There is also potential selection bias, since recruitment for our study was based on the group of patients available in one clinic only, which also limits external validity. What is more, self-reported measures that we used require good insight into one’s functioning and behavior, which can be distorted in clinical samples.

Our sample size was limited. The current sample size did not allow us to apply the Bonferroni correction for testing multiple mediation models. Specifically, not significant, but close to statistical significance (*p* = 0.089) moderation effect for distraction seeking needs further investigation in future research. Also, the limited sample size did not allow for verifying a more complex model of relationships between variables, including all coping styles and relationships between them in a single statistical model.

## 5. Conclusions

We conclude that the role of coping is recognized, but also needs future research, specifically as follows:

1. In the current study, the level of emotion-oriented coping was an important moderator for the relationship between the level of perceived stress and satisfaction with life. Specifically, the high level of emotional coping is associated with a greater impact the level of stress can have on lowering satisfaction with life;

2. In the current study, the female participants experienced a higher level of stress and lower satisfaction with life. The role of emotion-oriented coping can be especially important among female uniformed personnel;

3. The comparison between the current study and other studies conducted suggests that, depending on the current situation of participants, different coping mechanisms can be involved in the moderating effects, which calls for more research in the future investigating the issue for various samples. So far, one meta-analysis [38] revealed a small-to-moderate overall mean effect size regarding the positive relationship between coping flexibility and psychological adjustment;

4. Training aimed at coping strategies can be beneficial, as specific coping mechanisms can have buffering effects on the elevated level of stress and its harmful effects. Emotion regulation training, mindfulness, or cognitive restructuring could be helpful in reducing psychological mechanisms responsible for experiencing stress as particularly harmful;

5. From a policy perspective, these findings highlight the importance of routine psychological screening for stress and coping styles in uniformed services. Standardized intervention protocols, integrating resilience training, psychoeducation, and stress-coping workshops, could be embedded within occupational health frameworks. Early identification of personnel with elevated emotion-oriented coping may enable timely preventive measures.

## Figures and Tables

**Table 1:** Sociodemographic characteristics of the current sample.

Variable	Category	*n*	%	*M*	*SD*	*min*	*max*
Gender	Female	34	18.6	46.94	8.38	30	66
	Male	149	81.4	44.21	4.99	33	57
Education	Secondary	89	48.6	45.55	6.11	33	66
	Higher	94	51.4	44.17	6.12	30	60

*n*—number of participants; *M*—mean value; *SD*—standard deviation; *min*—minimum value; *max*—maximum value.

**Table 2:** Descriptive statistics for stress coping styles, the level of perceived stress, and satisfaction with life.

Variables	*M*	*SD*	*min*	*max*	*S*	*K*	*S-W*	*p*	α
Task-oriented style	53.60	11.26	13	75	−1.51	3.65	0.87	<0.001	0.88
Emotion-oriented style	42.60	11.47	10	66	−0.37	0.06	0.98	0.031	0.91
Avoidant style	42.10	9.89	16	62	−0.61	0.33	0.97	<0.001	0.83
Distraction seeking	19.30	5.13	8	31	−0.10	−0.59	0.99	0.073	0.76
Social diversion	14.80	4.25	2	23	−0.72	0.50	0.96	<0.001	0.75
Perceived stress	19.30	6.38	5	37	−0.02	−0.30	0.99	0.293	0.88
Satisfaction with life	21.00	5.49	5	33	−0.19	−0.28	0.99	0.065	0.76

*M*—mean value; *SD*—standard deviation; *min*—minimum value; *max*—maximum value; *S*—skewness; *K*—kurtosis; *S-W*—Shapiro–Wilk test for normality; *p*—statistical significance; *α*—Cronbach’s α reliability coefficient.

**Table 3:** Results of correlation analysis.

Variables	1.	2.	3.	4.	5.	6.	7.
1. Task-oriented style	--						
2. Emotion-oriented style	0.362 **	--					
	0.229; 0.482						
3. Avoidant style	0.495 **	0.286 **	--				
	0.376;. 597	0.147; 0.414					
4. Distraction seeking	0.199 **	0.252 **	0.867 **	--			
	0.055; 0.335	0.111; 0.383	0.826; 0.899				
5. Social diversion	0.640 **	0.153 *	0.837 **	0.489 **	--		
	0.545; 0.718	0.008; 0.292	0.788; 0.876	0.370; 0.592			
6. Perceived Stress	−0.045	0.573 **	−0.132	−0.059	−0.214 **	--	
	−0.190; 0.101	0.466; 0.663	−0.272; 0.014	−0.203; 0.087	−0.349; −0.071		
7. Satisfaction with Life	0.103	−0.284 **	0.162 *	0.082	0.214 **	−0.426 **	--
	−0.048; 0.250	−0.417; −0.140	0.012; 0.305	−0.069; 0.230	0.065; 0.353	−0.541; −0.294	
8. Age	−0.064	−0.140	−0.059	−0.040	−0.027	0.028	−0.120
	−0.207; 0.083	−0.280; 0.005	−0.202; 0.088	−0.184; 0.106	−0.171; 0.119	−0.117; 0.173	−0.265; 0.031

* *p* < 0.05; ** *p* < 0.01.

**Table 4:** Mean values of analyzed variables acquired in the group of female participants and in the group of male participants.

	Females		Males						
Variables	*M*	*SD*	*M*	*SD*	*t*	*df*	*p*	*d*	Effect Size
Task-oriented style	53.59	12.63	53.64	10.97	−0.03	180	0.980	−0.01	Small
Emotion-oriented style	44.32	9.31	42.25	11.91	0.95	180	0.343	0.18	Small
Avoidant style	41.15	10.35	42.34	9.80	−0.63	180	0.528	−0.12	Small
Distraction seeking	18.76	4.85	19.37	5.20	−0.62	180	0.535	−0.12	Small
Social diversion	14.76	4.81	14.76	4.13	0.00	180	0.999	0.01	Small
Perceived stress	22.35	6.08	18.64	6.27	3.13	181	0.002	0.60	Medium
Satisfaction with life	18.78	6.34	21.50	5.17	−2.56	169	0.011	−0.50	Medium

*M*—mean value; *SD*—standard deviation; *t*—value of independent samples *t*-test; *df*—degrees of freedom; *p*—two-tailed statistical significance; *d*—Cohen’s *d* effect size measure.

**Table 5:** Analysis of coping styles in the role of moderators of relationships between the level of perceived stress and satisfaction with life.

Coping Style	Effect	*B*	*Z*	*p*	*R* ^2^
Task-oriented style	Perceived stress	−0.36 [−0.49; −0.22]	−5.39	<0.001	0.188
	Coping style	0.04 [−0.03; 0.12]	1.03	0.303	
	Moderation effect	−0.01 [−0.01; 0.01]	−0.15	0.881	
Emotion-oriented style	Perceived stress	−0.35 [−0.49; −0.20]	−4.84	<0.001	0.205
	Coping style	−0.03 [−0.10; 0.05]	−0.78	0.438	
	Moderation effect	−0.01 [−0.02; −0.01]	−2.35	0.019	
Avoidant style	Perceived stress	−0.37 [−0.50; −0.23]	−5.40	<0.001	0.195
	Coping style	0.05 [−0.03; 0.14]	1.17	0.242	
	Moderation effect	−0.01 [−0.02; 0.01]	−0.85	0.395	
Distraction seeking	Perceived stress	−0.38 [−0.51; −0.26]	−5.86	<0.001	0.196
	Coping style	0.04 [−0.10; 0.20]	0.56	0.579	
	Moderation effect	−0.02 [−0.04; 0.01]	−1.70	0.089	
Social diversion	Perceived stress	−0.33 [−0.47; −0.19]	−4.71	<0.001	0.197
	Coping style	0.17 [−0.01; 0.39]	1.70	0.089	
	Moderation effect	0.01 [−0.02; 0.04]	0.36	0.716	

*B*—regression coefficient; *Z*—test for regression coefficient’s significance; *p*—statistical significance.

## Data Availability

Data available at authors.
